# Generalized Pairwise Comparisons in Dose Optimization Oncology Trials: Beyond Safety to Multi-outcome Dose Selection

**DOI:** 10.1158/1078-0432.CCR-25-4590

**Published:** 2026-04-17

**Authors:** Emily Alger, Ruitao Lin, J. Jack Lee, Ying Yuan, Christina Yap

**Affiliations:** 1 Clinical Trial and Statistics Unit, Institute of Cancer Research, London, United Kingdom.; 2 The University of Texas MD Anderson Cancer Center, Houston, Texas.

## Abstract

**Purpose:**

The primary objective of dose-finding oncology trials (DFOT) is to determine the recommended phase II dose. Although dose selection has traditionally relied on clinician-reported safety outcomes, the goal of DFOTs increasingly focuses on the integration of safety, activity, and tolerability within decision-making, in line with modern dose optimization strategies. Seamless phase I/II designs often use decision frameworks to quantify trade-offs between outcomes to guide dose selection. However, assigning numerical values to reflect such trade-offs can be difficult as clinical judgments and interpretations often vary between investigators.

**Experimental Design:**

With stakeholders, including clinical teams and patients, potentially considering their own prioritization of outcomes, generalized pairwise comparisons, including the win ratio (WR), provide a statistical framework mirroring this clinical decision-making by evaluating treatment benefit against prioritized outcomes. Using the WR, doses are compared across prespecified prioritized outcomes sequentially, with the optimal dose yielding the largest proportion of favorable (winning) patient-pair comparisons. This article presents WIN-DOSE, a hierarchical, multi-outcome WR-based approach for dose optimization. We demonstrate the performance of WIN-DOSE in a two-arm randomized dose optimization trial incorporating dose-limiting toxicities for safety, preliminary response for activity, and both dose intensity and patient-reported outcomes for tolerability.

**Results:**

When one dose is clearly favorable, WIN-DOSE consistently identifies the optimal dose. We also demonstrate how the WR can accommodate different trade-offs between safety, activity, and tolerability, supporting transparent and clinically relevant dose selection.

**Conclusions:**

The WR can support transparent and clinically relevant patient-centric dose selection decision-making, aligning with the broader goals of early-phase DFOTs.

**Translational Relevance**

Although dose-finding oncology trials (DFOTs) have traditionally relied on clinician-reported safety outcomes, the growing prevalence of dose de-escalation in later-phase studies highlights the need for multi-outcome decision-making to more accurately identify optimal dosages. Generalized pairwise comparisons (GPC), including the win ratio (WR), offer a nonparametric framework that can incorporate multiple outcomes simultaneously into dose selection. In the DFOT setting, this approach is relevant for integrating safety, activity, and tolerability in dose decision-making. In line with research advocating for the broadening of tolerability assessment in clinical trials, the WR offers a means to incorporate preliminary efficacy assessment, patient-reported outcomes, dose modification data, and other clinically relevant endpoints, alongside traditional clinician-reported toxicity measures to strengthen dose selection. With the goals of DFOTs continuing to broaden, multi-outcome decision-making will gain increasing relevance. GPCs therefore represent a promising methodologic tool to support this evolution, facilitating the development of safe, active, and tolerable treatments for patients.

## Introduction

The primary goal of an early-phase dose-finding oncology trial (DFOT) is to identify recommended dose(s) for further investigation in subsequent trials, primarily assessing the safety, activity, and tolerability of a new treatment. Traditionally, such dose recommendations have relied upon the safety evaluation of a treatment using clinician-reported measures such as Common Terminology Criteria for Adverse Events (CTCAE) grading. Under a traditional chemotherapeutic paradigm where treatment efficacy increases with dose, DFOTs have often focused on the determination of a maximum tolerated dose (MTD). The MTD is identified as the highest dose safeguarding clinician-assessed safety, often defined in terms of the percentage of dose-limiting toxicities (DLT) experienced by patients on treatment (1). Although this approach is frequently adopted in practice, its limitations for safety and tolerability evaluation are well established, including the underreporting of subjective toxicities by clinicians and discordance between clinician- and patient-assessed toxicity reporting (2, 3).

Consistent with such research, growing evidence suggests that dose(s) recommended for further testing in DFOTs may be shown to be intolerable upon evaluation in later-phase trials or after regulatory approval (4). Retrospective reviews of the targeted cancer drug ibrutinib indicate that up to 34% of patients require dose modifications of the full treatment dose within routine clinical practice, primarily due to adverse events (5). Such findings have motivated the critical need for dose reduction trials (6), with preliminary findings indicating that lower doses of ibrutinib are more tolerable and may maintain therapeutic effectiveness (7).

The case studies of such targeted drugs demonstrate the vital role of dose optimization trials within a treatment’s clinical development pathway. The selection of lower, active treatment doses offers both tolerability and financial advantages compared with the selection of unnecessarily higher doses (8). Growing scientific interest in dose optimization paradigms is being driven by initiatives such as FDA Project Optimus (9), the Methodology for the Development of Innovative Cancer Therapies Taskforce (10), and Friends of Cancer Research (11).

With novel treatments no longer consistently exhibiting increasing activity with increasing treatment dosage (12), we should look beyond the determination of the MTD to identify optimal active and tolerable doses for further investigation. With this motivation in mind, many dose-finding trial designs have been proposed, which can be broadly classified into efficacy-integrated designs and two-stage designs (13). The efficacy-integrated designs incorporate a risk–benefit trade-off from the outset of the trial to guide dose assignment and selection. In contrast, two-stage designs first escalate doses based on safety and then randomize patients among multiple doses to identify the dose with the optimal risk–benefit profile. In this article, we focus on the second stage of these two-stage designs: the randomized dose optimization stage. The risk–benefit trade-off in these designs may focus on toxicity and activity assessments (14, 15) or may incorporate patient tolerability by integrating patient-reported outcomes (PROs) (16–19). Statistically formalized trade-offs specify how these outcomes interact with one another within interim and final decision-making and may include loss-based (16) or utility-based approaches (20, 21). The existing multi-outcome dose-finding designs using risk–benefit trade-offs have primarily been developed to guide interim adaptive decision-making and final dose selection (22). Designs such as EffTox (23) and the BOIN suite (14, 20) support joint toxicity–efficacy decision-making at interim analyses, using binary or ordinal endpoints to escalate, de-escalate, or stay at the same dose. For practical application (24, 25), these designs rely on the specification of a quantitative utility function to support decision-making throughout the trial. However, eliciting appropriate trade-offs between outcomes for these approaches is often challenging, with numerical utilities potentially unintuitive, complicated, and varying subjectively between investigators, particularly when multiple endpoints or multilevel endpoints are involved.

In practice, when recommending phase II dose(s), stakeholders such as clinical teams, patients, sponsors, and oversight committees may already be implicitly generating their own preferences between clinical endpoints by considering the traditional aims of DFOTs. Accordingly, Porter and colleagues (26) have proposed ranked efficacy–toxicity outcome criteria to replace the elicitation of design parameters within adaptive dose selection trials. This approach takes inspiration from methodologic research on generalized pairwise comparisons (GPC) (27, 28). Since their introduction, GPC measures, such as the win ratio (WR), have been utilized to evaluate endpoints composed of hierarchical prioritized outcomes, with particular prominence in cardiology (29). GPC analysis has been proposed as a statistical framework to support the evaluation of less intensive treatment regimens, in which efficacy and tolerability are often jointly considered (30, 31). Correspondingly, within a DFOT setting, GPCs have the potential to provide a comparison between patient outcomes observed at two (or more) dose levels, recommending doses that maximize the number of patients with favorable outcomes according to a hierarchy of endpoints.

Although Porter and colleagues take inspiration from GPCs to inform a decision criterion, the associated methodology relies on a statistical backbone modeling each outcome. Such model-based trial designs are reliant on the available resources, training of statisticians, and interpretation by principal investigators. In contrast, a key advantage of GPC measures is that they are nonparametric and do not require the statistical modeling of marginal outcomes or their complex interdependencies. The flexible framework can instead formalize and statistically reflect clinical and patient-centric decision-making often adopted in practice to recommend doses while effortlessly integrating an ever-increasing number and level of outcomes within decision-making. To reflect such decision-making, we use GPC measures to compare two doses and ask the following question: “Which of these treatments are (i) safe, (ii) active, and (iii) tolerable?”

In this article, we present WIN-DOSE, a WR-based trial design to support multi-outcome decision-making. In doing so, we demonstrate how GPCs can broaden the assessment of safe, active, and tolerable doses within a DFOT utilizing hierarchical endpoints at the final analysis, once all relevant data are available. Rather than guiding adaptive dose escalation, GPCs provide a structured approach for assessing the relative safety, activity, and tolerability of doses within DFOTs, thereby supporting dose selection at the end of the study. Although GPC measures include net treatment benefit, WRs, and success odds (32), we focus our investigation on the WR, a popular method to analyze hierarchical composite outcomes (33). A case study presents exemplar use of the WR for inferential decision-making within a two-arm randomized dose optimization trial comparing two doses. We illustrate decision-making using the WR across various scenarios and doses with toxicity, activity, and tolerability trade-offs in a simulation study. We then discuss key considerations and the role of WRs in supporting clinical decision-making in DFOTs.

## Materials and Methods

### WR analysis

The WR is an example of a GPC measure. Such measures make inferences using a hierarchy of prioritized endpoints, traditionally assessed in terms of “wins.” Suppose we are comparing two treatment doses, dose A and dose B, with n_A_ and n_B_ patients assigned to each dose, respectively. The WR compares the observed patient outcomes between n_A_ patients treated at dose A and n_B_ patients treated at dose B by assessing pairwise comparisons, which can be made between patients allocated to each dose.

Suppose outcomes are prioritized such that outcome 1 is the most important, outcome 2 is the second most important, outcome 3 is the third most important, and so on. With the application of WRs primarily focused on cardiovascular trials (33), exemplar hierarchies of endpoints have previously included (i) time to death, (ii) time to hemorrhagic stroke, and (iii) time to ischemic stroke (28). For each fixed patient pair, the observed outcomes of two patients on doses A and B are compared at outcome 1. A patient pair favors dose A (or “wins” for dose A) at this outcome if the patient receiving dose A has a more favorable observed outcome than the patient receiving dose B. When patients have identical observed outcomes at outcome 1, they are subsequently assessed for outcome 2. The pair is considered a “win” for the dose with the better outcome. Supposing both patients once again have equivalent observed outcomes, we now look at outcome 3 and so on. For patient pairs in which both patients have identical outcomes, this patient pair is labeled as a “tie.” This procedure is evaluated for all possible patient pairs. Denoting the total number of patient pairs favoring dose A as N_A_ and those favoring dose B as N_B_, the WR of dose B over dose A is defined as follows:

$$\text{WR}\;=\;N_{B}/N_{A}.$$

The WR estimate is ≥ 0. A WR <1 indicates that dose A has more winning patient pairs than dose B. A WR >1 indicates that dose B has more winning patient pairs than dose A.

In this article, we estimate the WR using unmatched comparisons (with n_A_ × n_B_ patient pairs), investigating every possible patient pairing between patients assigned to doses A and B. See the Supplementary Materials for other pairwise comparisons.

### Outcomes

For application within the DFOT setting, we ensure that our prioritization of endpoints aligns with the goals of an early-phase DFOT setting—namely, to assess the safety, activity, and tolerability of a new treatment. With safety and preliminary efficacy more commonly evaluated within DFOTs (34), this article places particular emphasis on the assessment of tolerability within DFOTs and its potential role in dose decision-making using the WR.

#### Broadening the assessment of tolerability within early-phase DFOTs

The limitations of traditional clinician-assessed safety reporting within trials have led to the redefining of treatment tolerability by the Friends of Cancer Research as “the degree to which symptomatic and nonsymptomatic adverse events associated with the product’s administration affect the ability or desire of the patient to adhere to the dose or intensity of therapy” (35).

Although safety and tolerability endpoints have traditionally used clinician-graded CTCAE assessment, Friends of Cancer Research broadens tolerability assessment to include the following three components:

(1) Clinician-reported outcomes, which may include traditional DLT assessment
(2) Case report data, which may include dose modifications, discontinuations, interruptions, patient hospitalization, and death
(3) Integration of the patient experience, particularly PROs (19, 35)

#### Selected outcomes for WR analysis

Within this article, WIN-DOSE identifies safe, active, and tolerable doses utilizing outcomes as per Table 1. This provides one exemplar hierarchy and other outcomes may also be considered in practice if deemed relevant.

**Table 1.** Outcomes and endpoints (an exampe hierarchy) utilized to estimate the WR in this article.

Outcome	Endpoint
Safety	(1) Occurrence of DLT, assessed by CTCAE grading (binary)
Activity	(2) Occurrence of treatment response (binary)
Tolerability	(3a) Occurrence of sufficient dose intensity (binary)
	(3b) Patient-reported tolerability using FACT-GP5 score (ordinal)

A DLT is defined as a clinician-assessed grade 3 or worse adverse event as per the CTCAE within the predefined short DLT observation window. Beyond serving as a measure of safety, this outcome also provides an initial indicator of treatment tolerability as per the Friends of Cancer Research tolerability definition (35). The preliminary response is defined using the traditional Response Evaluation Criteria in Solid Tumors version 1.1 criteria (36). To assess tolerability to treatment, we align the outcomes with the recommendations provided by Friends of Cancer Research (35). A measure of dose intensity summarizes the treatment received by a patient, accounting for dose modifications, discontinuations, interruptions, and patient hospitalization and death. We suppose patients receive sufficient dose intensity if they have received at least 80% of the overall intended dose. Finally, the PRO tolerability measure “I am bothered by side effects of treatment” assesses patients’ overall side effect impact (37). Alternative PRO tolerability concepts, such as symptomatic adverse events and overall health-related quality of life, may also be considered in line with OPTIMISE-ROR guidance for PROs in DFOTs (19). Such tolerability measures provide a more comprehensive assessment by capturing patient experiences outside the predefined DLT window.

#### Prioritization of outcomes

A trialist wishing to utilize the WR must consider the prioritization of outcomes for their specific application. The hierarchies can be chosen to reflect the priorities of multidisciplinary stakeholders. Aligned with the goals of a DFOT, an exemplar hierarchy of outcomes may consider (i) safety, assessed by the occurrence of DLT; (ii) activity, assessed by the occurrence of treatment response; (iii) treatment administration tolerability, assessed by sufficient dose intensity; and (iv) patient-reported tolerability, assessed by the FACT-GP5 score. This is an example of a four-layer WR, as comparisons between patient outcomes occur over four outcomes/layers, each defined by their corresponding trial endpoints (Table 1).

However, in a DFOT study, trialists may not consider the evaluation of DLT alone sufficient for decision-making. Rather, trialists may consider DLTs assessed jointly alongside the preliminary response to be more valuable evidence for decision-making. In instances in which the outcomes are equally important, the WR may be generalized to consider endpoints that, rather than single outcomes themselves, instead quantify a trade-off between outcomes.

Within this article, we subsequently consider the following hierarchy of outcomes: (i) trade-off between the occurrence of DLT and preliminary response, (ii) the occurrence of sufficient dose intensity, and (iii) FACT-GP5 score. We identify this as the three-layer WR.

Although many articles have proposed the use of numerical utilities to trade off safety and efficacy endpoints (20), as the WR identifies winners across discrete categories, the relative ranking of outcomes (rather than elicited numerical utilities) informs inference. This eliminates the need for investigators to assign numerical utilities, which may otherwise limit the application of utility-based dose decision-making criteria in practice (26).

In Table 2, we identify the rational rankings the trialists may use to trade off DLT and response in this setting. The rank prioritizing response is mirrored within the utility functions proposed by U-BOIN (20), with “No DLT + No response” having a lower value than “DLT + Response” (30 and 65, respectively).

**Table 2.** Rational rankings of DLT and response trade-off, with best and worst outcomes for each scenario marked.

Rank	Worst outcome	​	Second worst outcome	​	Second best outcome	​	Best outcome
Rank prioritizing safety	DLT + no response	<	DLT + response	<	No DLT + no response	<	No DLT + response
Rank with intermediate ties	DLT + no response	<	DLT + response	=	No DLT + no response	<	No DLT + response
Rank prioritizing activity	DLT + no response	<	No DLT + no response	<	DLT + response	<	No DLT + response

Across the different ranking approaches, the worst and best outcomes remain the same, and it is rather the ordering of the intermediate outcomes that differ. In a ranking that prioritizes safety, avoiding a DLT is valued more highly than achieving a response, whereas in a ranking that prioritizes activity, achieving a response is valued more highly than avoiding a DLT. The rank with intermediate ties considers a patient with a DLT and response equivalent to a patient with no DLT and no response.

**Figure 1.**
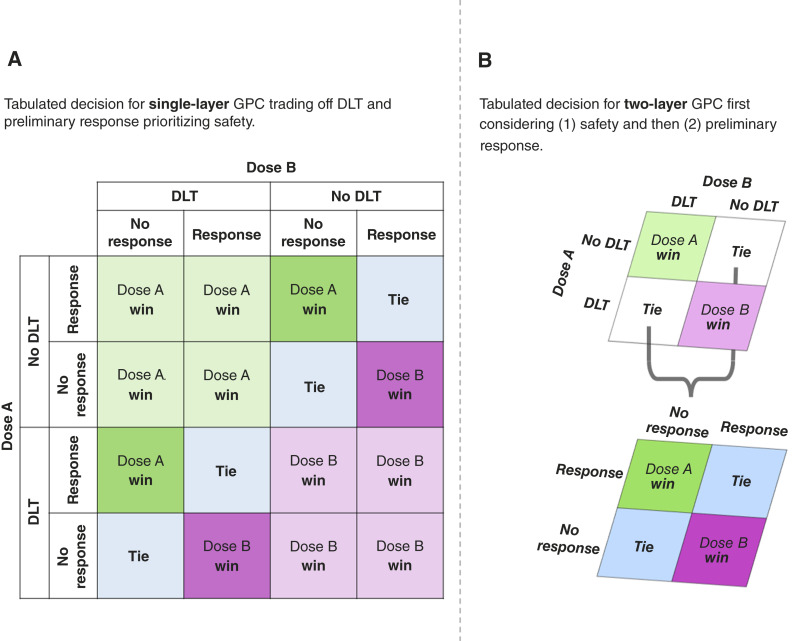
Illustrative demonstration of the equivalence between a GPC utilizing a trade-off between DLT and preliminary response prioritizing DLTs (**A**) and a GPC first assessing DLTs and then preliminary response (**B**). Color coding indicates boxes with an equal number of wins between the two approaches to indicate their equivalence.

Notably, there is a correspondence between the four- and three-layer WRs with rankings prioritizing DLTs and response. For example, a three-layer WR prioritizing DLTs is equivalent to the four-layer WR, which assesses patient pairs using DLTs first and then response. This is explained graphically in Fig. 1. Suppose one patient experiences a DLT and another does not. In this scenario, both the four- and three-layer WRs will reach the same “win” decision at their first outcome as no DLT is always favored over response for a rank prioritizing DLTs. If both patients have the same DLT outcome, the three-layer WR will still assess the patient’s response within its first layer, whereas the four-layer WR will defer the comparison to the subsequent response layer. Although the layer at which the “win” is determined may differ, the final decision remains the same. Similarly, the three-layer WR prioritizing response is equivalent to a four-layer WR prioritizing response first and then DLTs. However, there is no similar equivalence for a ranking with intermediate ties.

### Simulation study

#### Simulation configuration

To demonstrate decision-making for DFOTs utilizing the WR, we present a simulation study. We utilize the WR within the final analysis of a two-arm randomized dose optimization trial to determine the optimal dose between the two doses, dose A and dose B. The analysis is completed in R version 4.2.1 using the “BuyseTest” package (38).

The WRs are calculated using the three-layer WR approach, which is equivalent to the four-layer WR approach for specific trade-offs. The trials are simulated with 30 patients on each dose arm. Both DLT and preliminary response are simulated as binary endpoints. A patient’s dose intensity is simulated as a continuous proportion of the full treatment dose and dichotomized at 80% to indicate sufficient or insufficient treatment dose. The PRO scores are simulated as an ordinal endpoint as per FACT-GP5. The DLTs are simulated using a Bernoulli distribution. Patients who experience a DLT are presumed to discontinue treatment and thus do not receive sufficient dose intensity over the course of treatment.

A Gaussian copula links PROs with dose intensity such that patients with greater side effect bother are associated with a greater likelihood of treatment modification or discontinuation (39). As such, patients with greater side effect bother are more likely to receive insufficient dose intensity over the course of the trial. A Gaussian copula further links PROs and DLTs such that patients experiencing a DLT are more likely to report greater side effect bother compared with those who do not experience a DLT. The DLT and response are correlated using a Gaussian copula, with a very mild correlation between the two endpoints investigated in the main article and a strong correlation investigated in the Supplementary Materials, indicating that patients with a DLT are more likely to respond to treatment.

#### Illustrative trial example: applying the WR in a dose optimization trial

We now present a hypothetical trial example illustrating an application of the WR within a DFOT setting and its potential role in determining a recommended phase II dose inspired by a phase I to II trial of sitravatinib and nivolumab in clear-cell renal cell carcinoma (40). Wishing to investigate doses of 60, 80, 120, and 150 mg of sitravatinib in combination with nivolumab, the original trial used a late-onset efficacy–toxicity design (41), utilizing DLT and preliminary efficacy for formal decision-making. The analysis at the end of the trial indicated two doses with near-identical utility. The trialists accordingly introduced a new criterion to compare these doses, with the Bayesian posterior probabilities of objective response rate, progression-free survival, and PROs considered in turn to compare doses and inform the recommendation of the phase II dose. A 120 mg dose of sitravatinib was recommended accordingly, with a favorable objective response rate, progression-free survival, and higher quality of life. The WR can formalize this approach by evaluating each endpoint by priority (as this trial evaluated response rate, survival, and PROs in turn) before recommending an optimal dose in a transparent and systematic way.

Table 3 presents the simulated data for this case study. In this example, although dose A is favored from a safety and tolerability perspective, dose B is favored in terms of preliminary efficacy.

**Table 3.** Simulated data for illustrative case study.

​	DLT rate (%)	Efficacy rate (%)	Sufficient dose intensity (%)	FACT-GP5 score (%)				
				Not at all	A little bit	Somewhat	Quite a bit	Very much
Dose A	27	80	87	16	23	37	18	7
Dose B	42	88	62	6	3	27	38	27

**Figure 2.**
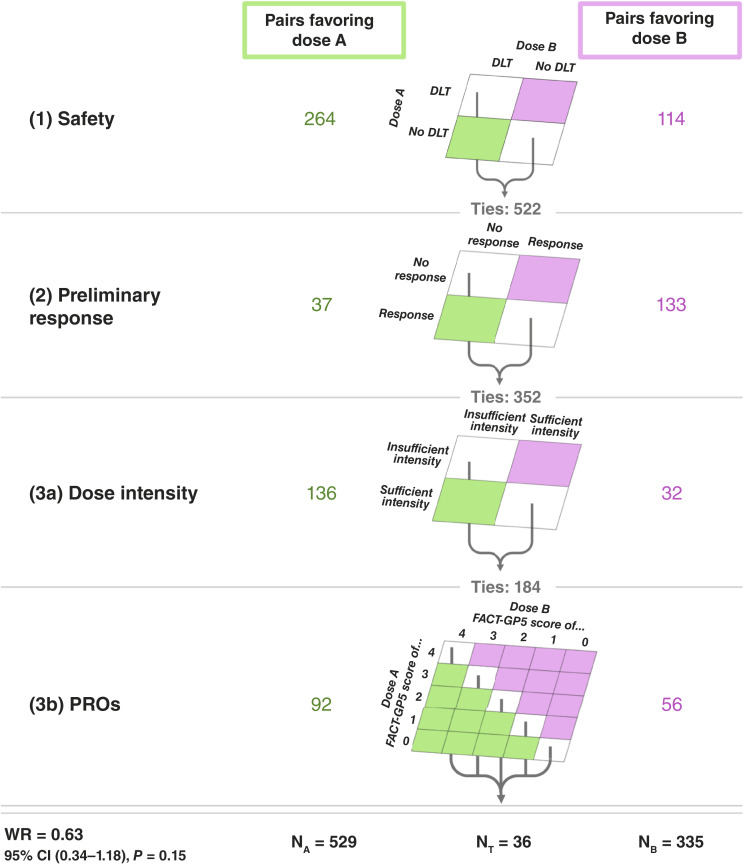
Hypothetical case study using the WR within a simulated clinical trial. WR is presented alongside the 95% confidence interval (CI) and *P* value of a two-tailed hypothesis test. N_A_, N_T_, and N_B_ indicate the number of favorable pairs for dose A, tied pairs, and the number of favorable pairs for dose B, respectively.

Supposing 30 patients receive either dose A or dose B in a two-arm randomized dose optimization trial using a ranking prioritizing DLTs, we utilize the WR at the final analysis to determine the best dose. Figure 2 follows this hypothetical trial and displays the number of favorable pairs and ties for each prioritized outcome. The figure is presented using the four-layer approach for ease of interpretation; however, as noted previously, this is equivalent to a three-layer approach with a DLT–response trade-off prioritizing DLTs. For tying pairs, the carryover of pairs from one layer to the next slowly decreases as the WR process unfolds. As expected, dose A has more favorable pairs for safety and tolerability endpoints, whereas dose B has more favorable pairs for the preliminary response endpoint. Once all 900 pairs have been evaluated, dose A has 529 favorable pairs, and dose B has 335 favorable pairs. The calculated WR of 0.63 (95% confidence interval, 0.34–1.18) indicates that dose A has the greatest number of patients with favorable outcomes. The *P* value of 0.15 from the two-tailed hypothesis test should be evaluated against a prespecified significance level to assess whether the estimate is deemed statistically significant. The Supplementary Materials provide details on the calculation of *P* values for the WR estimand.

## Results

**Figure 3.**
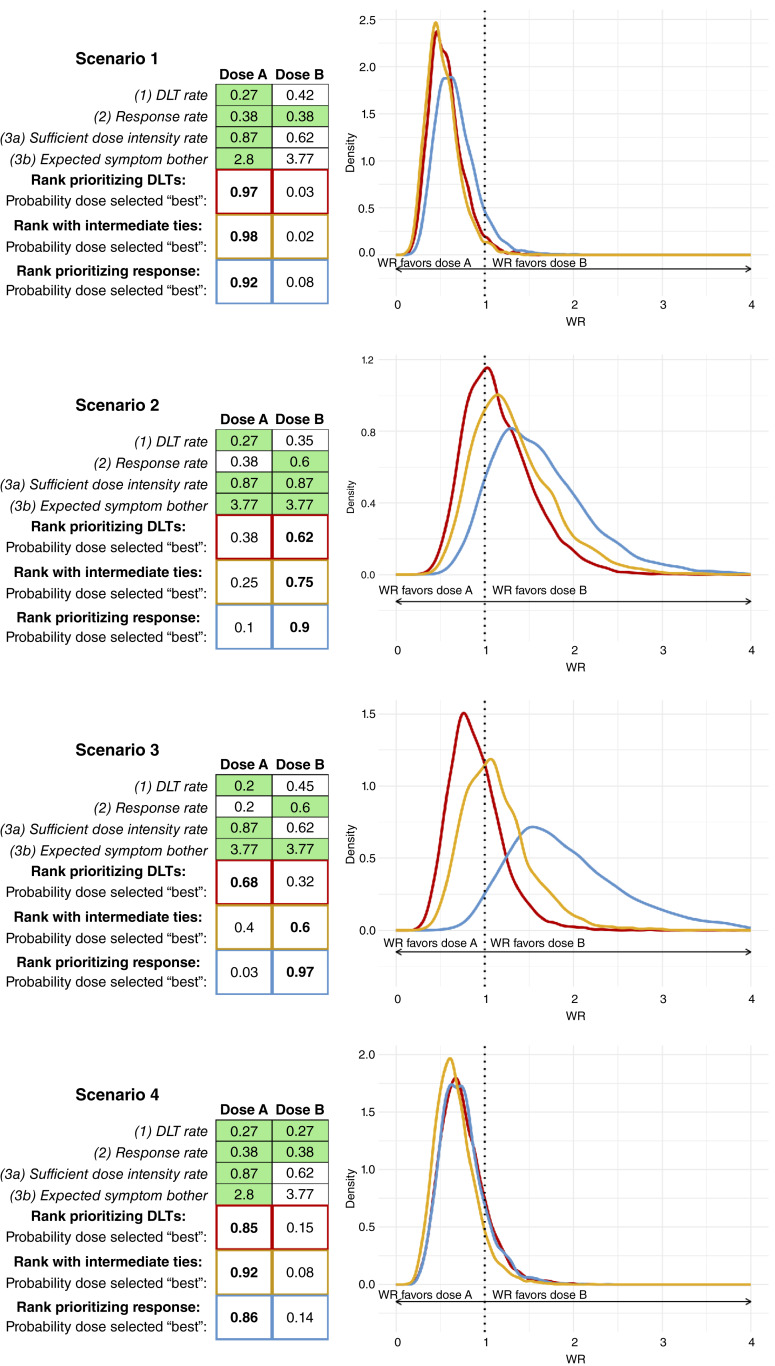
(Left) One of the four simulation scenarios, where the empirical probability of dose A or dose B is identified as the best dose by the WR across 5,000 simulations when DLT and response rate are very mildly correlated using a Gaussian copula (with a correlation of 0.1). Green is used to highlight the favored dose associated with each endpoint, and bold indicates the dose most often recommended as best for each ranking of DLT and preliminary response. (Right) A density plot displaying the distribution of WR estimates across 5,000 simulations for each scenario.

We evaluate WIN-DOSE for 5,000 clinical trial simulations across four scenarios (Fig. 3). The simulation scenarios are designed to reflect contemporary toxicity, efficacy, and tolerability patterns observed in oncology trials (12, 22). These include scenario 1, in which higher doses are associated with greater toxicity but similar response (40, 42). Scenarios 2 and 3 reflect situations in which both toxicity and response increase with dose (43), both with and without an impact on tolerability (44). Scenario 4 illustrates a setting in which toxicity and response are comparable across doses, but tolerability is worse at the higher dose (45).

In scenario 1, we observe how the WR behaves when dose A is the preferred dose for each outcome. Scenarios 2 and 3 indicate performance when different doses have the best outcomes, and scenario 4 assesses the impact of including tolerability endpoints alone in decision-making.

In scenario 1, regardless of safety response ranking, the WR correctly recognizes dose A as the best at least 92% of the time. Scenario 2 illustrates that the dose best in terms of the highest priority outcome may not necessarily be identified as the optimal dose by the WR. Although dose B has an 8% higher DLT rate than dose A (0.35 vs. 0.27), its 22% higher response rate (0.60 vs. 0.38) ensures that the WR hierarchies recommend dose B more often than dose A. Because the difference in DLT risk is modest, approximately 57% of pair comparisons looking at DLTs first result in a tie and are subsequently evaluated using response. Dose B, therefore, wins most tiebreaks due to its markedly better response despite its less favorable DLT profile. As a result, the safety-focused rank still identifies dose B as superior 62% of the time, whereas a WR prioritizing response identifies dose B as the best 90% of the time, with a ranking with intermediate ties lying between these values. From a clinical perspective, this scenario illustrates how a modest increase in toxicity may be outweighed by a larger improvement in preliminary efficacy, leading WIN-DOSE to favor the more active dose.

In scenario 3, although dose A has advantageous safety and tolerability outcomes, dose B shows a significantly better preliminary response rate. Unlike scenario 2, the dose intensity now also favors dose A. Utilizing an efficacy-focused rank (prioritizing response), the WR identifies dose B as the best dose 97% of the time. However, the WR, using a safety-focused rank (prioritizing DLTs), instead recommends dose A more often, 68% of the time. A rank with intermediate ties shows some preference for recommending dose B although this ranking utilizes more DLT data within decision-making, showing some interest in recommending dose A. This scenario illustrates that rather than clearly favoring one dose over another, the WIN-DOSE may distribute its support across doses. Generally, a favorable first outcome for one dose over another is not, on its own, sufficient for the WR to favor that dose—the magnitude of the difference across outcomes is also critical in decision-making.

In scenario 4, we demonstrate the role of tolerability endpoints in decision-making. Supposing both doses have equally good safety and efficacy endpoints, the WR confidently recognizes that dose A has better dose intensity and PRO metrics.

Supplementary Table S1 of the Supplementary Materials presents the mean number of patient pairs favoring dose A, dose B, and tied pairs. As expected, a rank with intermediate ties has the greatest number of ties across each scenario, but the number of ties remains low (at most 9.1% of all assessed patient pairs). Butler and colleagues (46) have cautioned against the interpretation of the WR in the presence of a high proportion of ties. With ties not featuring in the computation of the WR (and yet representing an absence of a win), utilizing the WR in such instances may overinflate the estimate of treatment effect.

Scenario 3 shows the greatest divergence in WR estimates for the three ranks trading off DLTs and preliminary response, reflecting the greatest divergence in the mean number of pairs favoring doses A and B. When ranked by prioritizing DLTs, dose A is recommended. Across 5,000 simulations, when intermediate ties are prioritized, a mean of 65 patient pairs shifts to favor either dose B or a tie. A mean of 157 patient pairs that favor dose A using a rank prioritizing DLT instead favor dose B when the ranking prioritizes response.

In line with the research linking adverse events to clinical benefit (47), additional simulations presented in Supplementary Fig. S1 of the Supplementary Materials evaluate the WR’s dose selection decision-making when patient-level DLT and preliminary efficacy responses are strongly correlated across the same simulation scenarios as per Fig. 3. The impact of this patient-level correlation is most influential in dose selection decision-making for scenario 3, in which the selection of dose A increases by 15% when a safety-focused WR rank is used to inform the final dose selection. Supplementary Table S2 of the Supplementary Materials presents the mean number of patient pairs favoring dose A, dose B, and tied pairs when DLT and response rate are highly correlated.

## Discussion

GPCs provide a framework to extend multi-outcome statistical dose selection criteria in DFOTs by assessing a hierarchy of endpoints, mirroring strategies utilized in clinical decision-making in complex settings. Although this article has primarily focused on the introduction of the WR within DFOTs, other GPC measures may be considered for their application within dose selection decision-making (28).

When a specific dose performs best across all outcomes, the WR consistently selects it as the optimal dose. In other cases in which doses are favored for different outcomes, the WR demonstrates its own implicit utility function, identifying which dose is best dependent on the hierarchy of prioritized endpoints. By incorporating outcomes that broaden the assessment of tolerability in DFOTs, the WIN-DOSE highlights the role of these outcomes and provides a structured, preplanned quantitative framework for dose selection, ensuring that recommended doses are not only safe and active but also tolerable.

Although many multi-outcome dose-finding trial designs depend on the modeling of patient outcomes, the WR provides a simple, nonparametric alternative that requires no statistical modeling for dose selection. This key advantage enables all stakeholders, including investigators and patients, to play a pivotal role in defining the outcomes and their hierarchical prioritization within dose selection. What is more, when using the WR, complex dependencies between outcomes need not be parametrically modeled, nor must utilities be explicitly assigned. In light of growing interest in dose optimization paradigms and as DFOTs wish to evaluate a growing number of outcomes, GPCs may become increasingly relevant and statistically advantageous. With WIN-DOSE, the outcomes included, and their prioritization within the hierarchy, are specified on a trial-by-trial basis. While the exemplar hierarchy in this article prioritizes safety, activity, and tolerability, trialists may also choose to incorporate other endpoints where relevant (e.g., pharmacokinetic/pharmacodynamic data). Further discussion on the impact of endpoint dependencies on WR estimation is provided in the Supplementary Materials.

The WIN-DOSE, in principle, supports the introduction of many outcomes within decision-making due to its prioritized hierarchy. In this article, we highlight the equivalences between WRs evaluating single outcomes in each layer and those incorporating trade-offs between outcomes within the same layer. Unlike existing designs that utilize utility or loss functions to trade off endpoints, WIN-DOSE does not require numerical elicitation of utility from investigators. Instead, the WR’s simpler need for a hierarchy of endpoints may help remove inter-rater subjectivity from dose decision-making and reduce the sensitivity of trial results (26).

Within its current methodologic framework, GPCs are principally suited to final analysis decision-making for dose selection, irrespective of whether the analysis arises within a dose escalation or a randomized dose optimization trial. Analysis considerations for applying the WR in practice are provided in the Supplementary Materials.

### Inference using the WR: What dose is best?

Owing to their small sample sizes, although analysis within DFOTs is often exploratory rather than statistically powered, correctly identifying an optimal dose has critical relevance for the continued investigation of the treatment within later-phase trials.

In Fig. 1, we demonstrate how WRs may be used as part of hypothesis testing for statistical inference. Acknowledging the small sample sizes of DFOTs, significance levels should be prespecified by trialists to assess statistically significant differences in WRs to support the evaluation of nonconfirmatory conclusions. In this article, we apply a straightforward criterion to identify the optimal dose by assessing whether the WR is greater than or less than 1. However, future work may wish to consider other potential criteria to identify the best dose, which may include an equivalence region around 1 in which both doses are deemed equally optimal.

Although we present scenarios illustrating dose selection decision-making using the WR under contemporary paradigms within this article, trialists wishing to use GPC analysis in dose selection decision-making should still carefully consider the mechanism of action of their prospective investigational treatment. In certain classes of agents, toxicity and efficacy may be only weakly correlated, whereas in others, the dependency may be moderate or strong. Accordingly, when evaluating the operating characteristics of the design, simulation studies should be undertaken to evaluate treatment-specific paradigms, including plausible correlations between toxicity and efficacy. Although the WR may be used for *ad hoc* WR analysis to evaluate inferences under different hierarchies of clinical priorities (48), such analysis should clearly be described as exploratory. It should not be modified after the trial to indicate a statistically significant result (46).

In addition, as the WR is a nonparametric method, it is more robust but less efficient than parametric and utility-based methods. When the parametric model assumptions hold, explicit modeling among various outcomes can yield more efficient methods. However, with multidimensional, complex, interdependent outcomes, it can be very challenging to formulate parametric models that capture the nature and patterns of all outcomes. Although GPC methods may be less efficient, they enjoy the advantages of being simple and robust.

### Existing limitations and future research directions of the WR for use in DFOTs

Although the methodology for computing the WR reflects multidisciplinary stakeholders’ prespecified prioritization of outcomes, inference using the WR may not always reflect how stakeholders collectively judge the most appropriate dose. The WR carries its own implicit utility for dose selection, determining how trade-offs across toxicity, response, and tolerability are weighted within the pairwise comparison. Future research should focus on understanding and eliciting the preferences of these stakeholders and on developing best practices for translating those preferences into a prespecified hierarchy and defining the granularity of outcome categories to capture real-world dose selection decision-making.

The performance of the WR depends on how the priority hierarchy is specified, which can be challenging in some applications. The WR reflects a coarse, implicit, step-function-like trade-off among endpoints, making it difficult to account for their relative importance as precisely as a utility-based approach. Although the hierarchy allows the WR to incorporate multiple endpoints, its gatekeeping property (i.e., stopping the evaluation of lower-priority endpoints once one dose shows more wins than the other at the current hierarchy) may lead to overlooking important information at lower levels. This may conflict with the totality-of-evidence principle outlined in the FDA’s dose optimization guidance. As such, before performing a GPC analysis, doses should be assessed against prespecified admissibility criteria across all relevant endpoints. Any dose deemed inadmissible (e.g., due to unacceptable toxicity or PRO burden) is removed and excluded from the subsequent hierarchical comparison. In this way, GPC is applied only to acceptable doses, ensuring that unfavorable outcomes that may occur for endpoints positioned lower in the WR hierarchy are not overlooked.

In this article, we have introduced the use of GPCs within a two-arm randomized dose optimization trial. However, dose optimization trials may wish to explore more than two doses. Although three-way comparisons between treatments using a WR have been explored (49), future work is needed to assess the suitability of these approaches for DFOTs. Future research could also explore extensions of GPC methodologies for their implementation in adaptive early-phase designs (including dose escalation) to inform interim decision-making and sample size determination (50).

As multi-outcome decision-making becomes increasingly important in DFOTs, methods such as the WR offer a valuable alternative to both *ad hoc* approaches and more complex model-assisted or model-based designs for guiding dose selection. The WR can sequentially evaluate safety, activity, and tolerability according to prespecified priorities informed by both clinical judgment and patient perspectives. As such, the WR supports patient-centric dose selection decisions that are transparent, clinically relevant, and aligned with the broader goals of early-phase DFOTs, ultimately supporting the development of therapies that are safe, active, and acceptable to patients.

## Supplementary Material

Supplementary Data Supplementary materials for manuscript

## Data Availability

The code and data presented in this article are publicly available in the following GitHub repository: https://github.com/alemily100/win_ratio.
