# Supplementary material for: Generalized Pairwise Comparisons in Dose Optimization Oncology Trials: Beyond Safety to Multi-outcome Dose Selection
Source: Clin Cancer Res. 2026 Apr 17;32(13):2787–96. doi: 10.1158/1078-0432.CCR-25-4590 (PMC13320202; doi:10.1158/1078-0432.CCR-25-4590)
Supplement: Supplementary Data — Supplementary materials for manuscript [file ccr-25-4590_supplementary_data_suppsm1.pdf]

Generalised pairwise comparisons in dose-optimisation oncology trials: beyond safety to multi-outcome dose selection

Supplementary materials

Pairwise comparisons for the WR

There are a number of pairwise comparisons which can be used to compare patients using the WR. Matched pairwise comparisons compare patients with similar characteristics as per a matching criterion, including age, sex, etc, with $m_A=m_B$ comparisons where m_A and m_B are the number of matched-pair patients in Dose A and Dose B, respectively. Stratified matching stratifies patients dependent on specified characteristics, with an unmatched comparison approach taken to compare patients within that stratum.

Calculation of p-values for the Win Ratio

Ozenne et al. present a methodology for calculating p-values for the Net Benefit estimator in the presence of complete and right-censored data based on U-statistic theory.[1] This framework may be directly applied to the Win Ratio, which admits a non-degenerate U-statistic under comparable conditions.[1]

Let $\widehat{\Delta}$ denote the U-statistic estimator of the Win Ratio estimand Δ with corresponding closed-form variance estimator $Var(\widehat{\Delta})$ as derived by Ozenne et al.[1] In line with standard U-statistic theory, $\widehat{\Delta}$ is asymptotically normally distributed.

Statistical inference is conducted via a Wald test of the null hypothesis,

$$H0: \Delta=0 \text{ versus } H1: \Delta \neq 0 .$$

The test statistic is given by

$$Z = \frac{\widehat{\Delta}}{\sqrt{Var\,(\widehat{\Delta})}},$$

which converges in distribution to a standard normal random variable under the null hypothesis. The two-tailed p-value is therefore computed as $p = 2\big(1 - \Phi(|Z|)\big)$ where $\Phi(\cdot)$ denotes the cumulative distribution function of the standard normal distribution.

This methodology is implemented in the R package *BuyseTest*.[2]

Supplementary simulations

		Pairs favouring Dose A	Pairs favouring Dose B	Tied pairs
**Scenario 1**	Rank prioritising DLTs	561	302	37
	Rank with intermediate ties	564	286	50
	Rank prioritising response	526	337	37
**Scenario 2**	Rank prioritising DLTs	401	442	56
	Rank with intermediate ties	364	455	82
	Rank prioritising response	334	510	56
**Scenario 3**	Rank prioritising DLTs	464	398	38
	Rank with intermediate ties	399	431	70
	Rank prioritising response	307	555	38
**Scenario 4**	Rank prioritising DLTs	501	360	40
	Rank with intermediate ties	517	332	51
	Rank prioritising response	501	359	40

Table S1 Mean number of pairs favouring Dose A, Dose B or tied across four scenarios with three rankings of DLTs and preliminary response across 5,000 simulation scenarios when DLT and response rate are very mildly correlated using a Gaussian copula (with correlation of 0.1). The green shading indicates the dose supported by the majority of pairs.

Scenario 1

	Dose A	Dose B
(1) DLT rate	0.27	0.42
(2) Response rate	0.38	0.38
(3a) Sufficient dose intensity rate	0.87	0.62
(3b) Expected symptom bother	2.8	3.77
**Rank prioritising DLTs:** Probability dose selected 'best':	**0.99**	0.01
**Rank with intermediate ties:** Probability dose selected 'best':	**1**	0
**Rank prioritising response:** Probability dose selected 'best':	**0.95**	0.05

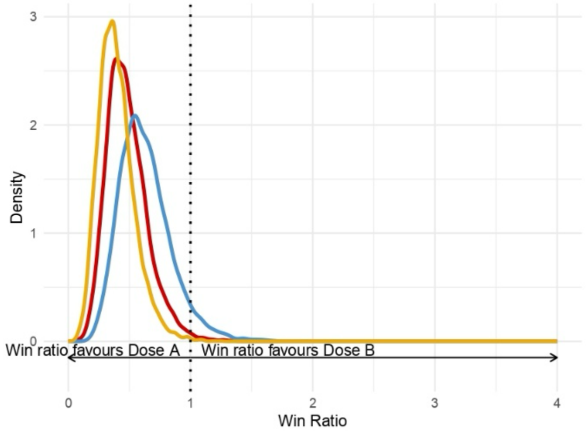

Scenario 2

	Dose A	Dose B
(1) DLT rate	0.27	0.35
(2) Response rate	0.38	0.6
(3a) Sufficient dose intensity rate	0.87	0.87
(3b) Expected symptom bother	3.77	3.77
**Rank prioritising DLTs:** Probability dose selected 'best':	0.43	**0.57**
**Rank with intermediate ties:** Probability dose selected 'best':	0.24	**0.76**
**Rank prioritising response:** Probability dose selected 'best':	0.06	**0.94**

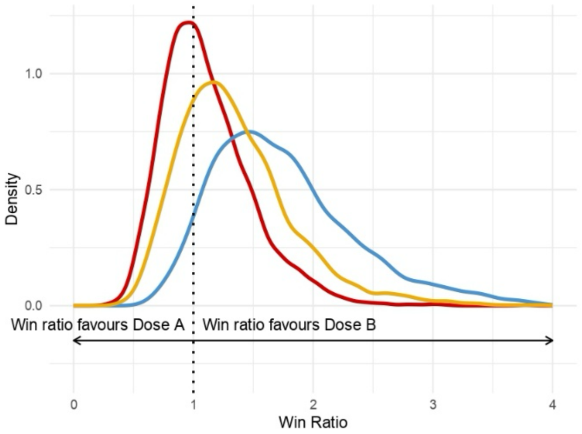

Scenario 3

	Dose A	Dose B
(1) DLT rate	0.2	0.45
(2) Response rate	0.2	0.6
(3a) Sufficient dose intensity rate	0.87	0.62
(3b) Expected symptom bother	3.77	3.77
**Rank prioritising DLTs:** Probability dose selected 'best':	**0.83**	0.17
**Rank with intermediate ties:** Probability dose selected 'best':	0.48	**0.52**
**Rank prioritising response:** Probability dose selected 'best':	0.01	**0.99**

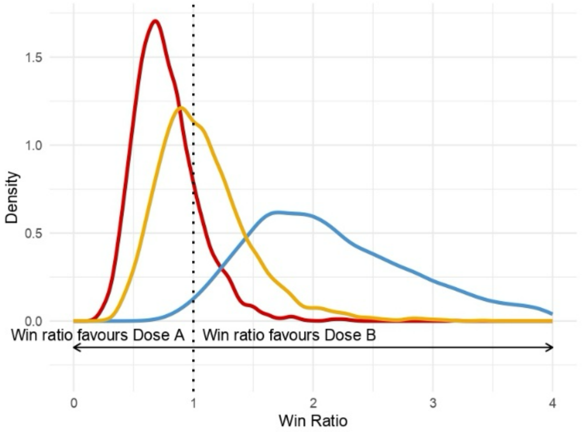

Scenario 4

	Dose A	Dose B
(1) DLT rate	0.27	0.27
(2) Response rate	0.38	0.38
(3a) Sufficient dose intensity rate	0.87	0.62
(3b) Expected symptom bother	2.8	3.77
**Rank prioritising DLTs:** Probability dose selected 'best':	**0.89**	0.11
**Rank with intermediate ties:** Probability dose selected 'best':	**0.98**	0.02
**Rank prioritising response:** Probability dose selected 'best':	**0.9**	0.1

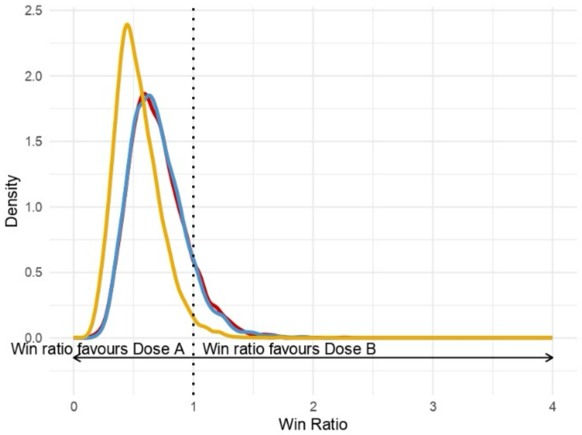

Figure S1 Left-hand side panels indicate one of four simulation scenarios and the empirical probability dose A or dose B is identified as the best dose by the WR across 5,000 simulations when DLT and response rate are strongly correlated using a Gaussian copula (with correlation of 0.7). Green is used to highlight the favoured dose associated with each endpoint and bold indicates the dose most often recommended as best for each ranking of DLT and preliminary response. Right hand side panels provide a density plot displaying the distribution of WR estimates across 5,000 simulations for each scenario.

		Pairs favouring Dose A	Pairs favouring Dose B	Tied pairs
**Scenario 1**	Rank prioritising DLTs	584	271	46
	Rank with intermediate ties	597	227	76
	Rank prioritising response	532	322	46
**Scenario 2**	Rank prioritising DLTs	405	428	67
	Rank with intermediate ties	346	438	116
	Rank prioritising response	315	518	67
**Scenario 3**	Rank prioritising DLTs	489	360	51
	Rank with intermediate ties	394	402	103
	Rank prioritising response	276	573	51
**Scenario 4**	Rank prioritising DLTs	510	343	47
	Rank with intermediate ties	546	281	74
	Rank prioritising response	511	342	47

Table S2 Mean number of pairs favouring Dose A, Dose B or tied across four scenarios with three rankings of DLTs and preliminary response across 5,000 simulation scenarios when DLT and response rate are strongly correlated using a Gaussian copula (with correlation of 0.7). The green shading indicates the dose supported by the majority of pairs.

<u>Supplementary discussion</u>

Whilst dependencies between endpoints need not be parametrically modelled when utilising the WR, this is not to say dependencies between endpoints do not play a *role* in estimation of the WR. GPC measures can inherently account for these associations between endpoints within their analysis.[3] As demonstrated in Scenario 3 (Figure 3 and Figure S1 of the Supplementary Materials), varying the patient-level association between DLT and response can alter the empirical probability of selecting Dose A, depending on the strength of the correlation. Suppose a two-layer WR evaluates prioritised endpoints (1) toxicity and (2) response. If toxicity and response are highly positively correlated, patients who respond are also more likely to have a DLT. These patients therefore may not contribute to the response comparison, because their toxicity outcome determines the win-loss assessment before response is evaluated. As such, the WR estimate in this setting may differ from another setting where overall toxicity and response rates are identical, but toxicity and response observations occur independently at the patient level.

Analysis considerations for applying the WR

Whilst the WR does not require the elicitation of numerical utilities, its application still depends on the prioritisation of outcomes in the form of a pre-specified hierarchical decision-making structure. This requires input from multidisciplinary stakeholders, including clinicians, statisticians, and patient representatives to ensure the ordering reflects clinically meaningful priorities and is appropriate for decision-making in early phase oncology trials. Careful consideration of how each outcome is measured is also vital. A measure with finer categories may capture more distinctions between patients than a broader measure. For example, consider the layer assessing safety. Whilst we consider a binary outcome at this layer (occurrence of DLT), we could instead consider a patient's maximal CTCAE grade (ranging from no toxicity, Grade 1, to Grade 5). Previously, there were four possible outcomes for a patient pair: (No DLT, No DLT), (No DLT, DLT), (DLT, No DLT), and (DLT, DLT). However, with a finer measure using the whole range of CTCAE grade to assess safety, there are 36 possible combinations, increasing the likelihood that a patient pair will favour one dose over the other based solely on safety, before considering other outcomes. In practice, using the full CTCAE range may be of limited utility, as distinctions between low-grade toxicities (e.g., Grade 1 vs 2) may have little clinical relevance for dose selection, yet they could disproportionately influence the WR.

Thus, trialists must consider how to measure each outcome, with an outcome measured continuously potentially further reducing the number of patients assessed for subsequent outcomes. In such instances, trialists may wish to utilise clinically relevant thresholds to define favourable outcomes.[4] The granularity of outcome categories can affect dose-selection decisions, highlighting the value of simulation studies to explore the impact of different measure selections on statistical inference.

Such considerations will also impact the number of ties at the end of the trial. As well as overinflating estimated treatment effect in presence of ties, [5] the WR cannot be interpreted as an odd. In such instances,

trialists may wish to consider other GPC measures such as the success odds which includes ties within their computation and can be interpreted as odds to aid clinical interpretation.[6] Other GPC measures may be employed if trialists foresee analysis will lead to a large number of ties.[6]

WR in presence of missing data and intercurrent events

Whilst this simulation study supposes we collect all patient outcomes by the time of analysis, in practice, intercurrent events such as death and dose discontinuation may result in only partial data being available at analysis. Such missing data may impact estimation of the WR.[7] With its implementation in randomised controlled trials with time-dependent survival endpoints, estimation of the WR with missing data has primarily focussed on censored data, with recommendations including the comparison of outcomes over common durations of observation.[4] Utilising such approaches may support the inclusion of time-to-event outcomes such as overall survival and progression-free survival within dose-selection decision-making, alongside other activity endpoints such as preliminary response. However, given that patients enrolled in early-phase trials often have a median survival of nine months,[8] future work providing recommendations for the handling of missing data in WR analyses in DFOTs is particularly critical.

References

1. Ozenne B, Budtz-Jørgensen E, Péron J. The asymptotic distribution of the net benefit estimator in presence of right-censoring. *Statistical Methods in Medical Research*. 2021;30(11):2399-2412. doi:10.1177/09622802211037067
2. *Buysetest: Implementation of the generalized pairwise comparisons. R package version 3.3.4*. 2025.
3. Buyse M, Saad ED, Peron J, et al. The net benefit of a treatment should take the correlation between benefits and harms into account. *Journal of Clinical Epidemiology*. 2021/09/01/ 2021;137:148-158. doi:https://doi.org/10.1016/j.jclinepi.2021.03.018
4. Verbeeck J, De Backer M, Verwerft J, et al. Generalized pairwise comparisons to assess treatment effects: Jacc review topic of the week. *Journal of the American College of Cardiology*. 2023/09/26/ 2023;82(13):1360-1372. doi:https://doi.org/10.1016/j.jacc.2023.06.047
5. Butler J, Stockbridge N, Packer M. Win ratio: A seductive but potentially misleading method for evaluating evidence from clinical trials. *Circulation*. 2024/05/14 2024;149(20):1546-1548. doi:10.1161/CIRCULATIONAHA.123.067786
6. Dong G, Huang B, Verbeeck J, et al. Win statistics (win ratio, win odds, and net benefit) can complement one another to show the strength of the treatment effect on time-to-event outcomes. *Pharmaceutical Statistics*. 2023/01/01 2023;22(1):20-33. doi:https://doi.org/10.1002/pst.2251
7. Li H, Chen W-C, Lu N, Tang R, Zhao Y. The elusiveness of the win ratio parameter in the presence of missing data. *Therapeutic Innovation & Regulatory Science*. 2024/05/01 2024;58(3):431-432. doi:10.1007/s43441-024-00645-2
8. Wheler J, Tsimberidou AM, Hong D, et al. Survival of patients in a phase 1 clinic. *Cancer*. 2009/03/01 2009;115(5):1091-1099. doi:https://doi.org/10.1002/cncr.24018
